# Successful resection of a solitary metastatic liver tumor from prostate cancer 15 years after radical prostatectomy: a case report

**DOI:** 10.1186/s40792-017-0292-4

**Published:** 2017-01-25

**Authors:** Hironari Kawai, Hiroaki Shiba, Masaru Kanehira, Taro Sakamoto, Kenei Furukawa, Katsuhiko Yanaga

**Affiliations:** Department of Surgery, The Jikei University School of Medicine, 3-25-8, Nishi-Shimbashi, Minato-ku, Tokyo 105-8461 Japan

**Keywords:** Metastatic liver tumor, Prostate cancer, Hepatic resection

## Abstract

**Background:**

A solitary metastatic liver tumor of prostate cancer is extremely rare because liver metastasis occurs as a part of systemic dissemination of prostate cancer. We herein report a successfully resected case of a solitary metastatic liver tumor from prostate cancer almost 15 years after radical prostatectomy.

**Case presentation:**

A 70-year-old male who had undergone radical prostatectomy for prostate cancer 15 years previously presented to our hospital for treatment of a liver tumor. Serum prostate-specific antigen was elevated at 13.77 ng/ml. Abdominal computed tomography revealed a solitary tumor with a diameter of 54 mm in segment 4 of the liver. No metastatic lesions were found in other organs. The patient was given a diagnosis of a metastatic liver tumor from prostate cancer, and he underwent medial segmentectomy. Microscopically, the resected specimen was composed of eosinophilic tumor cells with oval nuclei and prominent nucleoli, which exhibited a cribriform pattern and a fused glands pattern with positive prostate-specific antigen and prostatic acid phosphatase staining; these findings were compatible with metastatic prostate cancer. Other than portal thrombosis that required anticoagulation, the patient made a satisfactory recovery and was discharged on postoperative day 15.

**Conclusion:**

To the best of our knowledge, this is the first report describing successful resection of a solitary metastatic liver tumor from prostate cancer in the medical literature. In such a rare circumstance, hepatic resection for liver metastasis of prostate cancer seems justified.

## Background

A solitary metastatic liver tumor of prostate cancer is extremely rare, as liver metastasis from prostate cancer occurs through the portal system from lymph node metastasis or carcinomatosis [1]. We herein report a successfully resected case of a solitary metastatic liver tumor from prostate cancer almost 15 years after radical prostatectomy.

## Case presentation

**Fig. 1:**
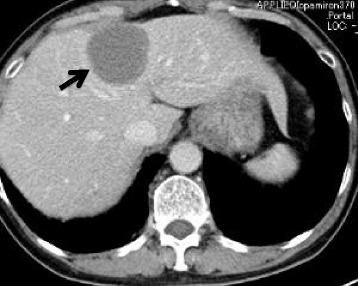
Enhanced computed tomography revealed a solitary, low-density, and hypovascular tumor with a diameter of 54 mm in segment 4 of the liver (*arrow*)

**Fig. 2:**
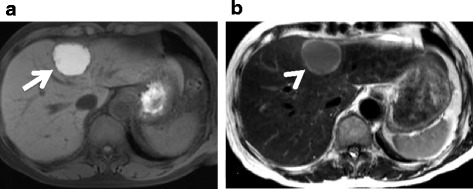
Magnetic resonance imaging revealed a high-intensity tumor on T1-weighted images (**a**; *arrow*) and a low-intensity tumor with surrounding high intensity on T2-weighted images (**b**; *arrowhead*)

**Fig. 3:**
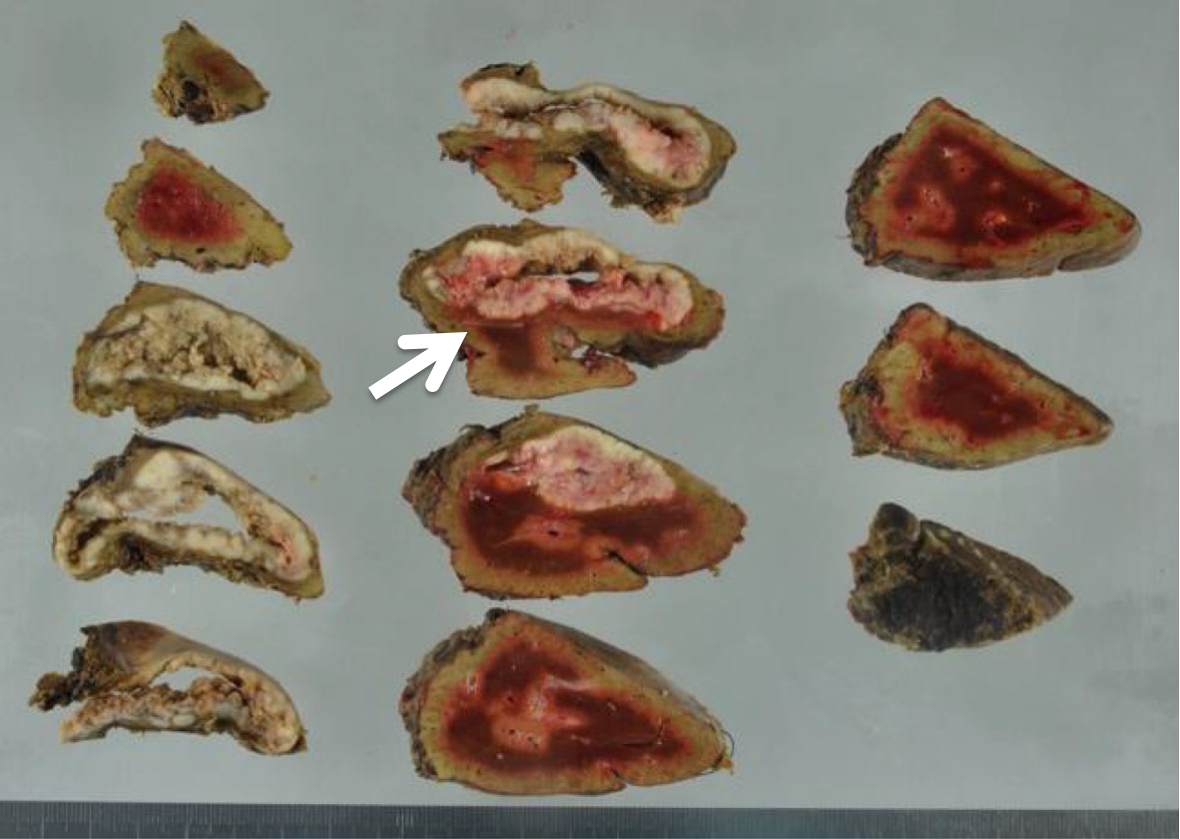
Macroscopic findings revealed a solid whitish hepatic tumor with central hemorrhage and necrotic changes, and the maximum diameter of the tumor was 55 mm (*arrow*)

**Fig. 4:**
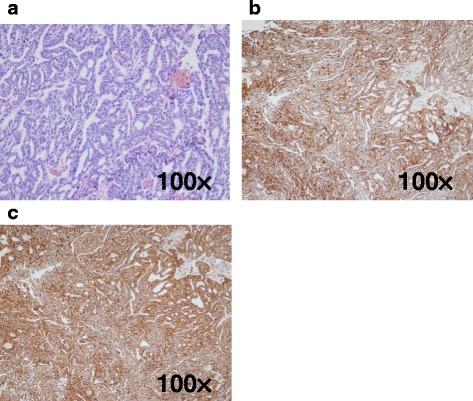
Microscopically, the resected specimen was composed of eosinophilic tumor cells with oval nuclei and prominent nucleoli, which exhibited a cribriform and fused glands pattern by hematoxylin-eosin staining (**a**) and stained positive for prostate-specific antigen (**b**) as well as prostatic acid phosphatase (**c**) (×100)

**Fig. 5:**
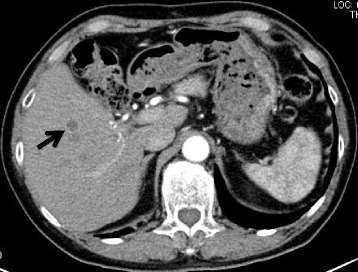
One year after hepatic resection, enhanced computed tomography revealed a solitary, low-density, and hypovascular tumor with a diameter of 10 mm in segment 5 of the liver (*arrow*)

A 70-year-old male who had undergone radical prostatectomy for prostate cancer 15 years and 1 month previously presented for treatment of a liver tumor. The patient had received adjuvant hormonal therapy for prostate cancer using goserelin acetate and bicalutamide. Eleven years after resection, local recurrence occurred, and the patient underwent 70 Gy of external radiation therapy; hormonal therapy was also switched at that time from bicalutamide to flutamide while goserelin acetate was continued. The local recurrence of prostate cancer was successfully treated and had a complete response. The patient had a past medical history of multiple arterial thromboses, including popliteal, femoral, and mesenteric artery thrombosis; he was maintained on 3.5 mg of warfarin per day. Laboratory evaluation revealed that serum prostate-specific antigen (PSA) was elevated at 13.77 ng/ml. Enhanced computed tomography (CT) revealed the presence of a solitary, low-density, and hypovascular tumor with a diameter of 54 mm in segment 4 of the liver (Fig. 1). Magnetic resonance imaging demonstrated a liver tumor with a high intensity on T1-weighted images (Fig. 2a) and a low intensity with surrounding high intensity on T2-weighted images (Fig. 2b) in the same area. No metastatic lesions were found in other organs on positron emission tomography-CT. With the diagnosis of a solitary metastatic liver tumor from prostate cancer, the patient underwent medial segmentectomy of the liver. Macroscopic findings of the resected specimen revealed a solid whitish tumor with maximum diameter of 55 mm with central hemorrhagic and necrotic changes (Fig. 3). Microscopically, the resected liver tumor was compatible with metastatic prostate cancer (Fig. 4). The patient developed portal vein thrombosis on postoperative day 6, which was successfully treated with anticoagulation. Otherwise, the patient made a satisfactory recovery and was discharged on postoperative day 15. Serum PSA decreased to 0.54 ng/ml after hepatic resection. Nine months after hepatic resection, serum PSA increased to 6.99 mg/ml, and enhanced CT at 1 year post-hepatic resection revealed a recurrent tumor in segment 5 of the liver (Fig. 5). The patient has received docetaxel chemotherapy for recurrent liver metastasis of prostate cancer.

## Conclusions

In 2013, prostate cancer was the most frequently diagnosed cancer in males (1.4 million) worldwide, while the incidence is still low in developing countries [2]. In 2008, approximately 14% of prostate cancer worldwide was diagnosed within the Asia-Pacific region, with three out of every four being diagnosed in Japan (32%), China (28%), or Australia (15%) [3]. The bone (90%), lung (46%), and liver (25%) are well-known and common metastatic sites of prostate cancer [1]. In general, liver metastasis occurs as a part of systemic dissemination of prostate cancer [4]. Therefore, solitary liver metastasis of prostate cancer is extremely rare. Batson et al. referred to the importance of the vertebral venous system (Batson’s plexus) as a metastatic pathway of prostate cancer. The vertebral venous system with their rich, valveless ramifications and connections by-passes the portal system, so this system offers a possible reason why a solitary metastatic prostate cancer occurred. To the best of our knowledge, this is the first report describing successful resection of a solitary metastatic liver tumor from prostate cancer in the medical literature. Although the utility of hepatic resection for patients with liver metastases from colorectal cancer or endocrine tumors has been established, for patients with non-colorectal, non-endocrine liver metastases, it remains unclear because of the limited numbers of patients in each primary tumor groups [5]. Adam et al. reported 5-year overall survival rates of patients with liver metastases from urologic cancer after hepatic resection of 66% in adrenal, 55% in testicular, and 38% in renal cancer, respectively; furthermore, these authors report that these patients may benefit from hepatic resection [5]. Herein, we also discuss portal vein thrombosis (PVT) after hepatectomy which is relatively rare complication. Kuboki et al. reported that the incidence rate of PVT after hepatectomy was 2.1% and that right-side hepatectomy, caudate lobectomy, splenectomy, and postoperative bile leakage were independent risk factors for PVT after hepatectomy [6]. Although this patient had no risk factors for PVT after hepatectomy, he had a past medical history of unidentified arterial thromboses, so this might have caused PVT. Hepatic resection for a solitary metastatic liver tumor from prostate cancer is one possible therapeutic option, provided that the systemic metastatic work-up is negative. In such a rare circumstance, hepatic resection for liver metastasis of prostate cancer seems justified.

## Abbreviations

CT: Computed tomography
PSA: Prostate specific antigen
